# Time Series Forecasting Model Based on the Adapted Transformer Neural Network and FFT-Based Features Extraction

**DOI:** 10.3390/s25030652

**Published:** 2025-01-23

**Authors:** Kyrylo Yemets, Ivan Izonin, Ivanna Dronyuk

**Affiliations:** 1 Department of Artificial Intelligence, Lviv Polytechnic National University, 79905 Lviv, Ukraine; kyrylo.v.yemets@lpnu.ua (K.Y.); i.izonin@bham.ac.uk (I.I.); 2 Department of Civil Engineering, School of Engineering, University of Birmingham, Birmingham B15 2TT, UK; 3 Faculty of Science & Technology, Jan Dlugosz University in Czestochowa, 42-200 Czestochowa, Poland

**Keywords:** time series, sensors, forecasting, ANN, transformer, fast Fourier transform, feature extraction, performance evaluation, attention mechanism, LSTM, DeepAR, deep learning, Big Data

## Abstract

In today’s data-driven world, where information is one of the most valuable resources, forecasting the behavior of time series, collected by modern sensor networks and IoT systems, is crucial across various fields, including finance, climatology, and engineering. However, existing neural network models often struggle with time series forecasting collected by different sensors due to challenges such as large data volumes, long-term dependencies, noise, and anomalies, which can negatively impact predictive accuracy. This paper aims to enhance the accuracy of time series forecasting by proposing an adapted transformer architecture combined with an innovative data preprocessing method. The proposed preprocessing technique employs the fast Fourier transform (FFT) to transition from the time domain to the frequency domain, enriching the data with additional frequency-domain features. These features are represented as complex numbers, which improve the informational content of the data for subsequent analysis, thereby boosting forecasting performance. Furthermore, the paper introduces a modified transformer model specifically designed to address the identified challenges in time series prediction. The performance of the proposed model was evaluated using three diverse datasets collected by different sensors, each with varying measurement frequencies, data types, and application domains, providing a comprehensive comparison with state-of-the-art models such as LSTM, FFT-LSTM, DeepAR, Transformer, and FFT-Transformer. Extensive evaluation using five distinct performance metrics demonstrates that the proposed model consistently outperforms existing methods, achieving the highest accuracy across all datasets.

## 1. Introduction

Forecasting time series is one of the most critical tasks in modern data analytics [1]. In an era of rapidly increasing information volumes and complex systems, the use of neural network techniques for this purpose is becoming increasingly popular [2]. The relevance of time series forecasting, particularly through neural networks in various application domains, stems from the need for accurate and timely predictions for resource management, load optimization, and cost reduction [3,4]. Deep learning neural networks are capable of effectively analyzing complex patterns in data [5], which enhances the forecasting of energy consumption, renewable energy production, and electricity prices [6,7]. This capability is especially crucial amid market volatility and changing climate conditions, where traditional methods may prove insufficient.

The application of neural networks opens new avenues for improving energy efficiency, enhancing management decisions, and ensuring the resilience of energy systems, especially using data collected by different sensors [8,9,10]. However, despite the potential advantages, this approach faces several significant challenges that require thorough examination [3,8,11].

First, the instability of the data on which forecasts are based presents a significant challenge. Time series, collected by different sensors, are often influenced by various factors, including seasonal fluctuations, trends, random variations, and changes in the external environment [12]. These factors can complicate the identification of patterns necessary for accurate predictions. For instance, in the energy sector, electricity consumption can vary significantly due to weather conditions, economic circumstances, and other social factors, rendering traditional models insufficiently effective.

Second, the demands for computational resources also pose a serious barrier. Training neural networks, especially on large datasets, can require substantial time and processing power [13]. Furthermore, the accuracy of forecasts derived from such data may not be adequate for specific application systems.

Choosing the right neural network architecture is another complex challenge [7,14]. There are numerous architectures available, including recurrent neural networks (RNN), long short-term memory networks (LSTM), and transformers, among others. Selecting the most suitable architecture for a particular task can be nontrivial. Such methods must account for data instability and large volumes of information, mitigate the risks of overfitting, and be reliable for practical applications.

Moreover, data preprocessing plays a crucial role in the effectiveness of the chosen artificial neural network model [15,16]. In recent years, methods for preprocessing time series data based on Fourier transforms have gained significant attention [17,18,19]. These methods involve transitioning from the time domain to the frequency domain, which provides more useful information and, consequently, improves the accuracy of predictions made using neural networks [20,21]. However, existing methods typically use only a single frequency characteristic, which may not be sufficiently informative for the selected neural network, leading to limited improvements in forecasting accuracy compared to the full potential of these approaches [22]. Therefore, the enhancement of existing methods and the development of new preprocessing techniques for time series data, tailored for analysis by artificial neural network architectures, will ensure a substantial increase in the accuracy of the forecasting process.

Consequently, this paper aims to develop a new method that synergizes FFT and neural network techniques, enabling the partial or complete mitigation of the aforementioned shortcomings of existing methods.

The main scientific contributions of this research can be formulated as follows:

- We developed a novel data preprocessing scheme for time series by replacing time components with the phase and amplitude of a complex number obtained using FFT. This approach facilitated the transition from the time domain to the frequency domain and enhanced the informational value for subsequent intelligent analysis of the time series using artificial neural networks;
- We investigated the effectiveness of a transformer architecture, which we adapted to tackle the problem of time series forecasting in conjunction with our proposed data preprocessing method based on FFT-based feature extraction across three different datasets;
- By comparing our results with a range of state-of-the-art approaches, we demonstrated an increase in forecasting accuracy based on various performance metrics for all examined datasets.

The practical value of the proposed approach lies in the following:

- Avoidance of the inefficiencies associated with processing long time series collected by modern sensor networks and IoT systems, enabling higher forecasting accuracy;
- Enhanced modeling of long-term dependencies through the attention mechanism embedded in the transformer architecture, allowing for effective handling of more complex time series without limitations on context length;
- Dynamic adaptability of the model to various types of time series is facilitated by both the attention mechanism and the transformer’s capability to efficiently process large datasets;
- Utilization of an extended range of frequency characteristics obtained from Fourier transform, allowing the retention of all frequency information while maintaining global context through the attention mechanism inherent in transformer architecture. This makes the proposed approach more flexible and suitable for a broader range of tasks that require working with long time series.

The article is organized as follows: In Section 2, a critical analysis of existing data preprocessing approaches for enhancing the accuracy of subsequent intelligent analysis of time series is presented. Section 3 contains information about the adaptation of transformer architecture for the task of time series forecasting, the fundamental principles of FFT specifically for data preprocessing, and a detailed description of the proposed approach that combines FFT and transformers to improve forecasting accuracy. Section 4 provides information about the three datasets used for modeling the proposed approach, details about the modeling procedure, and results based on various performance metrics for the three studied datasets. Section 5 includes a comparison of the proposed method with several state-of-the-art approaches that can be applied to the problem at hand. Conclusions based on the findings of the study are presented in Section 6.

## 2. State of the Art

Today, there are numerous data preprocessing techniques whose application can enhance accuracy and even reduce the duration of the training process for the selected model [23,24]. Among the most common methods, those belonging to the classes of normalization and standardization should be highlighted [25]. Such methods are used to bring data to a certain scale or distribution. This is especially important for neural networks, as they may not work efficiently with data that have varying scales. However, using such methods directly for time series leads to a situation where all features exert an equal influence on the training process. This can significantly affect the accuracy of forecasting such data [26].

Among time series preprocessing methods from another class, smoothing methods such as moving averages or exponential smoothing should be mentioned [27]. These are quite often used in the scientific literature to remove noise and identify key trends in time series. They are especially useful when a time series contains many random fluctuations. However, the use of methods that perform overly strong smoothing can lead to the loss of important information about short-term changes in the data [28]. Moreover, for some time series, smoothing may not be sufficiently accurate, as these methods do not account for more complex patterns. This can reduce forecasting accuracy, which limits their practical application.

Among the class of reliable and effective methods currently available, those based on the Fourier transform stand out [29]. Such methods allow for the identification of the main frequency components of a time series, enabling a neural network to better detect seasonal and cyclical patterns in the data. They offer several advantages over other classes of time series preprocessing methods [30,31]. In particular, by enabling a transition to the frequency domain of signal processing, it becomes possible to isolate important features that may be hidden in the time domain. In addition, these methods are well suited for analyzing cyclic time series, which are characteristic of many different processes. Let us consider the existing methods of this class in more detail.

There are several significant studies in this area demonstrating promising results. In particular, reference [21] proposed combining recurrent neural networks, specifically LSTM, with frequency transformations to improve time series processing. Additionally, reference [17] explored the potential of utilizing both time and frequency properties to enhance the performance of LSTM in classifying physiological signals. The results indicated improvements in both accuracy and training speed due to the introduction of frequency characteristics. This supports the hypothesis regarding the importance of incorporating frequency components in time series analysis tasks, especially for long and complex data sequences.

However, despite certain advancements in this field, there are limitations. For example, in one study [32], the ForeNet model employed a combination of Fourier transform and recurrent networks for forecasting. This approach focused primarily on complex weighted networks, which could complicate the application of the proposed method in real-world scenarios involving large datasets due to the intricacies of training and parameter tuning. While some of these approaches effectively handle frequency components, they do not address issues related to scalability and computational efficiency.

The study [33] presented an attempt to enhance the accuracy of forecasting financial time series through noise removal techniques such as wavelet transforms and by decomposing signals into spectra using Fourier transform. However, these methods encountered limitations when applied to different types of time series, with their effectiveness largely depending on the data specifics and computational costs.

In [34], an alternative approach was proposed that integrates neural network tools with Fourier transform. The authors suggested using deep neural networks with sinusoidal activation functions, where weights were initialized using the Fourier transform. This approach demonstrated improved generalization properties of the model through regularization and the application of sinusoidal functions to build simplified models. Although this method effectively extrapolates nonlinear trends, its limitations include a fixed architecture based on sinusoidal activation functions, which reduces flexibility when working with various data types.

The authors of [18] developed an approach that combines short-time Fourier transform (STFT) with LSTM for grasp-stability prediction tasks. The model showed promising results in predicting instability based on force and pressure data. However, a key limitation of this approach is that STFT is designed for short-term frequency transformations, which may be inadequate for more complex and longer time series.

To address the aforementioned limitations, the reference [19] proposed a combination of FFT and LSTM for forecasting mobile traffic in software-defined networking (SDN). In this approach, FFT was used to obtain the phase of a complex number, replacing the time series measurements with this feature. This method facilitated the transition from the time domain to the frequency domain when working with time series. The authors demonstrated that this approach significantly improved forecast accuracy while maintaining lower computational complexity compared to traditional methods such as ARIMA.

Despite these positive results, the proposed approach has several limitations. First, a major drawback is the use of LSTM, which, while capable of preserving long-term dependencies, struggles to effectively handle large time series or data with prolonged dependencies due to architectural constraints. Second, relying solely on the real part of the complex number does not provide the necessary informational richness for the artificial neural network, thereby reducing its potential for achieving even greater forecasting accuracy. This limitation is also evident in the work presented in [35], where only one frequency characteristic was utilized for the examined time series. In this paper, we aim to address the limitations of existing approaches by developing a new time series preprocessing method based on FFT, which enhances the informational value for subsequent analysis using artificial neural networks.

## 3. Materials and Methods

This section describes the key components and processes involved in adapting transformer architecture for the task of time series forecasting. It presents the fundamental derivations of the fast Fourier transform (FFT) and outlines the method for extracting two frequency characteristics from the time series, subsequently replacing each time series measurement with these characteristics. The proposed method for combining the FFT-based data preprocessing approach with the adapted transformer architecture is also detailed.

### 3.1. An Architecture of the Adapted Transformer for Addressing Time Series Forecasting Tasks

Transformer architecture has gained significant popularity in the field of machine learning, particularly in natural language processing, and it is applied to various tasks, such as translation and text analysis [36]. Due to their ability to efficiently process long sequences of data and identify relationships between elements over large distances, transformers are also used in other areas, including time series forecasting.

Transformers demonstrate notable advantages in tasks that involve multiple time series, such as weather forecasting. In this context, parameters with complex interrelationships—like temperature, humidity, and pressure—are often analyzed. The application of transformers allows for capturing these interdependencies, thereby improving forecasting accuracy [36].

Since the introduction of transformers in time series forecasting, several modifications have been developed. This is an actively researched area focusing on enhancing model capabilities for long-term predictions in real-world applications, such as energy, transportation, and resource planning. With their attention mechanism, transformers have shown significant potential in modeling complex dependencies.

The transformer architecture proposed in this work for time series forecasting involves significant modifications compared to the classical transformer architecture described in [36], which was specifically designed for natural language processing. This approach enhances its effectiveness for the task at hand.

**Figure 1:**
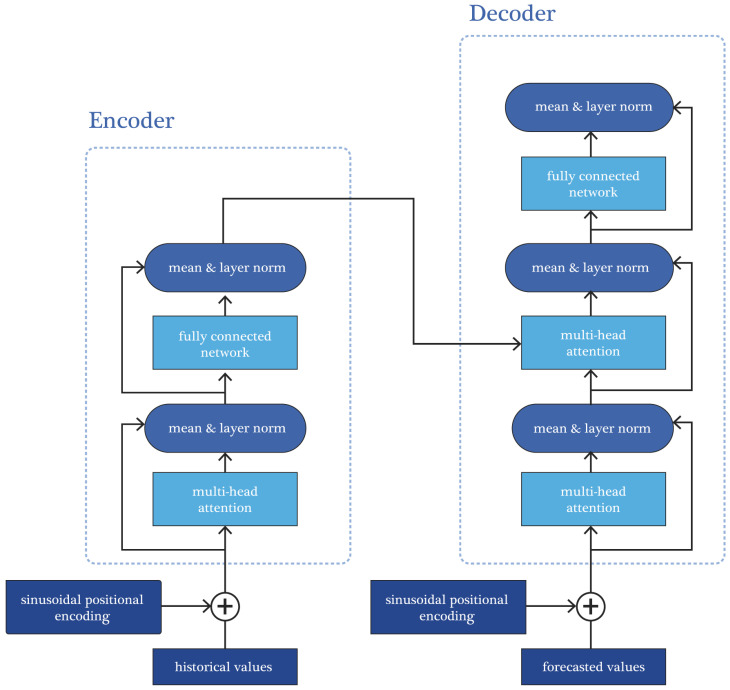
Flowchart of the adapted transformer architecture for time series forecasting task.

The flowchart of the proposed transformer architecture is shown in Figure 1. Components that have been replaced or added to the existing architecture [36] are highlighted in red.

One of the fundamental changes in the adapted architecture compared to the existing one is the removal of the tokenizer and embedding layer, which are traditionally used to convert input data into a format suitable for model processing. Additionally, this approach significantly simplifies the architecture and reduces its computational complexity, which is a crucial advantage when working with time series, where every optimization of resources matters.

Instead of the positional encoding used in the architecture from [36], this paper proposes the implementation of sinusoidal positional encoding, which is more natural for modeling time sequences. This type of encoding does not require additional learnable parameters and more effectively reflects the temporal order of the data. It particularly emphasizes the importance of the most recent elements in the sequence, which is critical for time series forecasting, where the order of events is essential for prediction accuracy.

Another significant change is the introduction of layer normalization instead of the conventional batch normalization. Batch normalization, typically applied in classical transformers, is often not optimal for time series data, as it relies on statistics from the entire data batch, potentially overlooking seasonal or cyclical changes. In contrast, layer normalization operates independently of batch size, providing model stability and better suitability for analyzing complex dynamic properties of time sequences.

All of these changes make the proposed architecture more flexible and resilient to various challenges encountered during time series analysis. The simplification of the model structure, along with the use of adapted techniques for positional encoding and normalization, not only reduces computational costs but also enhances overall forecasting accuracy. This allows for more efficient handling of large datasets, improving model performance in long-term forecasting tasks.

### 3.2. Fundamental Principles of FFT for Time Series Preprocessing Tasks

Fourier transform is a mathematical operation that transforms a function of time (or space) into a function of frequency [37]. It is an important tool in signal analysis that allows complex signals to be decomposed into simple harmonic components [38]. For continuous signals, this transformation is known as continuous Fourier transform.

The formula for continuous Fourier transform is as follows [35]:

(1) $$F(\omega )=\int_{-\infty }^{\infty }f(t)e^{-i\omega t},$$

where

f(t) is the time function (the original signal).

F(ω) is the frequency function (the Fourier transform).

ω is frequency (radians per second).

t is time.

Discrete Fourier transform (DFT) is applied to discrete signals and is used to convert a signal from the time domain to the frequency domain. Unlike continuous Fourier transform, the DFT deals with finite sequences of data, such as time series.

The DFT can be represented as follows [35]:

(2) $$X_{k}=\sum_{n=0}^{N-1}x_{n}e^{-i\frac{2\pi }{N}kn},k=0,1,2,...,N-1,$$

where

$X_{n}$ is the input sequence (discrete signal).

$X_{k}$ is the spectral coefficients after Fourier transform.

N is the number of points (length of the discrete sequence).

After applying the discrete Fourier transform, the result consists of complex numbers that contain information about the signal’s frequency components. A complex number can be visualized as a point on a coordinate plane, where each number has both a real and an imaginary part.

For further processing and transmission of these data to the model, complex numbers are often converted into polar form [35]. This means that instead of using the real and imaginary parts, each number is described by two parameters:

1. Magnitude (length of the vector)—defines the amplitude of the frequency component;
2. Argument (angle of the vector)—determines the phase of the frequency component.

For further processing and input into the model, complex numbers are often converted to polar form. This means that instead of using the real and imaginary parts, one can use the magnitude and angle.

A complex number, which is the result of Fourier transform, can be represented in polar form using the following formulas:

(3) $$\left|X_{k}\right|=\sqrt{Re{\left(X_{k}\right)}^{2}+Im{\left(X_{k}\right)}^{2}},$$

(4) $$arg\left(X_{k}\right)=arctan\left(\frac{Im\left(X_{k}\right)}{Re\left(X_{k}\right)}\right).$$

These quantities are used for further analysis and input into the model, as they provide a clearer representation of the signal’s frequency characteristics.

### 3.3. Proposed Time Series Forecasting Model Based on the Adapted Transformer Neural Network and FFT-Based Features Extraction

After transforming the time series using discrete Fourier transform, the obtained data are fed into the artificial neural network model. This study proposes to utilize both frequency characteristics of the time series in the form of components (3) and (4). Specifically, after applying Fourier transform to the studied time series, each time step is replaced with the phase and amplitude of a complex number. Consequently, the model receives an expanded input sequence that is twice the original length:

(5) $$t_{1},t_{2},...{,t}_{n}\to A_{1},\varphi_{1},A_{2},\varphi_{2},...{,A}_{n},\varphi_{n},$$

where

$t_{1},t_{2},...{,t}_{n}$ are points in the time domain.

$A_{1},A_{2},...{,A}_{n}$ are amplitudes obtained after discrete Fourier transform.

$\varphi_{1},\varphi_{2},...{,\varphi }_{n}$ are phases obtained after discrete Fourier transform.

This approach allows for further work solely with the frequency information of the signal, which, unlike existing methods, includes both (3) and (4).

The pseudocode for the training procedure of the proposed time series forecasting model based on the adapted transformer neural network and FFT-based features extraction scheme is shown in Algorithm 1.

**Algorithm 1** Training proposed model with FFT features$\mathrm{Input}:\;\mathrm{Time}\;\mathrm{series}\;\mathrm{data}\;\{t_{1},t_{2},...,t_{n}$}, m—window size, H—prediction horizon **1: for** i = 1 to n-H-1 **do** **2:**   $\mathrm{Extract}\;\mathrm{time}\;\mathrm{window}:\;\{t_{i},\;t_{i+1},\;...,t_{i+m-1}$} **3:**   $\mathrm{Compute}\;\mathrm{FFT}\;\mathrm{features}:\;\mathrm{fft}\_\mathrm{features}=\mathrm{FFT}([t_{i},\;t_{i+1},\;...,t_{i+m-1}$]) **4:**   Compute amplitude and phase: (amplitude_feat, phase_feat) = FFT_transform(fft_features) **5:**   Train model: $\mathrm{model}.\mathrm{train}(x=(\mathrm{amplitude}\_\mathrm{feat},\;\mathrm{phase}\_\mathrm{feat}),\;y=[t_{i+m},\;t_{i+m+1},\;...,t_{i+m+H-1}$]) **6: end for**


As shown in Algorithm 1, the fast Fourier transform (FFT) should be calculated only within the context of our model. The context size will vary depending on the task and dataset, and in our case, it ranges from 24 to 60 time units for our datasets. During the prediction phase, since we already have the actual values, there is no need to compute the reverse Fourier transform (i.e., the inverse FFT). For training, we simply calculate the FFT *n* times for *m* values, where *m* represents the context size, *n* corresponds to the length of the time series, and *H* corresponds to the prediction horizon of the model. This approach ensures that we efficiently utilize the frequency-domain features without unnecessary computational overhead while still maintaining the required information for model learning and prediction.

To demonstrate how the proposed preprocessing method for time series can be beneficial for further processing with transformer architectures utilizing the attention mechanism [36], it is important to consider how the model employs both temporal and frequency characteristics to enhance its performance.

The attention mechanism can be described as follows:

(6) $$Attention(Q,K,V)=softmax(\frac{QK^{T}}{\sqrt{d_{k}}}),$$

where Q are queries.

K is the keys.

V is the values.

$d_{k}$ is the dimensionality of the keys.

When the model receives only the amplitudes and phases of the frequency components, the transformer can easily process the frequency characteristics of the signal, simplifying the learning of important frequency dependencies.

Since we are working with a time series represented as frequency components (3) and (4), i.e., amplitudes and phases for each point, it is crucial that the queries, keys, and values in (6) take into account both the frequency features themselves and their context within the series. Specifically, each point $t_{i}$ may have its own frequency characteristics $A_{i}$ and $\varphi_{i}$, but their context will depend on the other points in the series. In this case, each point has its amplitude $A_{i}$ and phase $\varphi_{i}$, and the attention mechanism should consider the relationships between different frequency components. Here is how the formulas for Q, K, and V would look in this context:

(7) $$Q=W_{Q}\cdot \left|\begin{array}{cc} A_{1} & \varphi_{1}\\ \vdots & \vdots \\ A_{n} & \varphi_{n} \end{array}\right|,$$

(8) $$K=W_{K}\cdot \left|\begin{array}{cc} A_{1} & \varphi_{1}\\ \vdots & \vdots \\ A_{n} & \varphi_{n} \end{array}\right|,$$

(9) $$V=W_{V}\cdot \left|\begin{array}{cc} A_{1} & \varphi_{1}\\ \vdots & \vdots \\ A_{n} & \varphi_{n} \end{array}\right|,$$

where $A_{i}$ and $\varphi_{i}$ are the amplitude and phase for point; $W_{Q}$, $W_{K}$, and $W_{V}$ are the weight matrices for queries, keys, and values, respectively; n represents the length of the context for the time series.

Thus, the model learns to focus attention on specific frequency components by utilizing the amplitude and phase for each frequency component, enabling it to identify the underlying frequency dependencies of the signal. This enhances the accuracy of forecasting large time series when tackling various time series prediction tasks not only with transformer architectures but potentially with other neural network approaches as well.

## 4. Modeling and Results

This section provides a description of the three datasets used for the experimental studies, an explanation of the key metrics employed to evaluate the performance of the proposed model, and the actual results obtained from the described datasets.

### 4.1. Datasets Descriptions and Its Preprocessing

The modeling of the proposed model was carried out on three different publicly available datasets. This selection was motivated by the fact that the datasets encompass diverse time series with varying measurement frequencies and specific characteristics. In the following, we examine them in more detail.

The first dataset represents a very long daily series reflecting wind energy production in megawatts (MW). Data were recorded every 4 s, starting from 1 August 2019, with a total of 7,397,147 entries. This dataset was sourced from the Australian Energy Market Operator (AEMO) [39], ensuring high accuracy in the measurements of the sought value. The dataset required no preprocessing.

The second dataset contains 32,072 daily time series representing temperature observations and rainfall forecasts collected by the Australian Bureau of Meteorology for 422 weather stations across the country. The data span from 2 May 2015 to 26 April 2017. Given that the original dataset had missing values, these were replaced with zeros to maintain the integrity of the dataset. It is available in an open repository and can be downloaded from [40].

The third dataset consisted of a time series with minute-level measurements of wind energy production from 339 wind farms in Australia. These data were also downloaded from the AEMO platform and included series of 6345 measurements for each wind farm. This dataset is available in an open repository and can be downloaded from [41].

These datasets cover various aspects of energy production and climate observations, allowing for the testing of models under different measurement frequencies, data types, and application tasks, providing a comprehensive evaluation of the proposed model’s performance.

### 4.2. Performance Indicators

The evaluation of modeling results was conducted using a range of key metrics. These metrics quantitatively assess how accurately the model predicts values on the test data. Utilizing multiple metrics allows for a comprehensive evaluation of the model’s performance from different perspectives: accuracy, error scaling, mean squared deviations, and symmetric relative errors. Below, we examine these metrics in more detail [35,42].

The MASE (mean absolute scaled error) measures the accuracy of the model’s forecast by comparing it to the accuracy of a naive forecast based on the trend of the data:

(10) $$MASE=\frac{\frac{1}{H}\sum_{t=l+1}^{l+H}\left|y_{t}-{\hat{y}}_{t}\right|}{\frac{1}{l-1}\sum_{t=2}^{l}\left|y_{t}-{\hat{y}}_{t-1}\right|}$$

where $y_{t}$ is a true value.

${\hat{y}}_{t}$ is a forecasted value.

l is the lag of the naive model (typically 1).

H is the number of points to forecast.

This metric is scale-independent, allowing for comparisons between models across different datasets.

The SMAPE (symmetric mean absolute percentage error) is a modification of the classic MAPE that provides a symmetric assessment of relative errors. It takes into account the absolute values of deviations between forecasts and actual values, normalizing them by the sum of their magnitudes:

(11) $$SMAPE=\frac{100\%}{H}\sum_{t=1}^{H}\frac{\left|y_{t}-{\hat{y}}_{t}\right|}{\frac{\left|y_{t}\right|+\left|{\hat{y}}_{t}\right|}{2}}$$

Symmetric mean absolute percentage error helps assess the relative error of a model, independent of the scale of the data, with a symmetric weighting for overestimated and underestimated forecasts.

The MAE (mean absolute error) measures the average absolute magnitude of forecast errors without considering their direction. It reflects the average deviation of the forecast from the actual value, providing insight into the model’s accuracy:

(12) $$MAE=\frac{1}{H}\sum_{t=1}^{H}\left|y_{t}-{\hat{y}}_{t}\right|$$

The MAE is quite useful for evaluating models, as its result is interpreted in the same scale as the original data.

The MSE (mean squared error) assesses the average squared deviation of forecast errors. By squaring the errors, this metric gives greater weight to larger deviations, making it more sensitive to significant errors:

(13) $$MSE=\frac{1}{H}\sum_{t=1}^{H}{\left(y_{t}-{\hat{y}}_{t}\right)}^{2}$$

The mean squared error is particularly useful when one needs to identify how the model behaves with large deviations in its forecasts.

The RMSE (root mean squared error) is the square root of MSE, returning the result in the same units of measurement as the original data. This makes it easier to interpret in the context of the specific application:

(14) $$RMSE=\sqrt{\frac{1}{H}\sum_{t=1}^{H}{\left(y_{t}-{\hat{y}}_{t}\right)}^{2}}$$

The RMSE is also sensitive to large deviations, but it provides a more interpretable assessment of error in the original units. This metric offers a clear picture of the average magnitude of errors, placing greater emphasis on larger mistakes. This makes it useful for assessing not only the accuracy but also the stability of the model.

### 4.3. Modeling and Results for All Investigated Datasets

Table 1 summarizes the results of practical experiments for the three investigated datasets using the proposed time series forecasting model based on the adapted transformer neural network and FFT-based feature extraction.

It is important to note that one model was trained on N time series for Datasets 2 and 3, where N corresponds to the number of stations and wind farms, respectively. Instead of training a separate model from scratch for each individual time series, we opted for a more generalized approach. This method allows us to predict any time series within the dataset, even those that were not present during the initial training phase. Specifically, the model is designed to handle new time series, which could correspond to new stations or wind farms not included in the original dataset. This approach ensures that the model can effectively make predictions for time series with unknown IDs or new instances, offering a scalable solution for future data without retraining the model from scratch. For the MASE metric, naive prediction was calculated for each time series separately, while one model predicted all time series. This can result in the MASE metric being high.

## 5. Comparison and Discussion

To assess the effectiveness of the model proposed in this paper, we compared its accuracy with that of existing methods. Specifically, we utilized state-of-the-art models that are applicable to the task at hand:

1. The long short-term memory network (LSTM model) [43];
2. The fast Fourier transform–long short-term memory network (FFT-LSTM model) [22];
3. The DeepAR forecasting algorithm (DeepAR model) [44];
4. The transformers model, based solely on attention mechanisms (Transformer model) [36];
5. The transformers model adapted from [36] + FFT with one input frequency (FFT(1)-Transformer model) [35];
6. Naïve prediction model.

**Figure 2:**
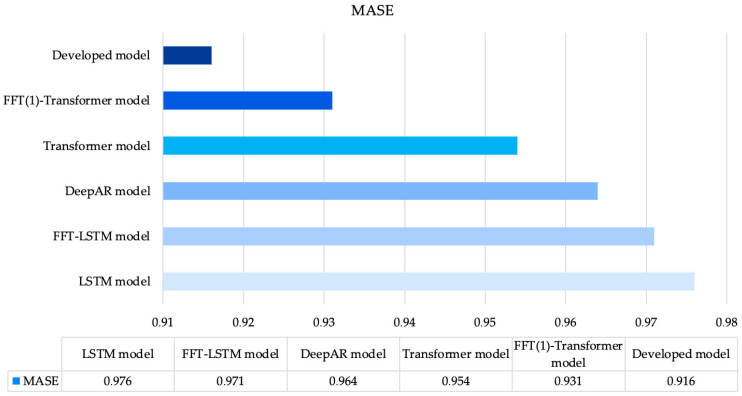
MASE values for all investigated models using Dataset 1.

Since the study involved modeling on three different datasets, we examine the comparison results for each dataset individually. Specifically, Figure 2 presents the accuracy comparison of all investigated models, illustrating MASE errors for the first dataset.

We also employed the Diebold–Mariano test to compare the predictive accuracy of two forecasting models. This test helps assess whether the models’ prediction performance differences are statistically significant. The criterion for this test was MSE. For the comparison, we used the developed model as the baseline and compared the forecasts of each model against it. The results of this test are shown in Table 2, Table 3 and Table 4.

Figure 2 shows that the LSTM model [43] exhibits the lowest accuracy among all the methods analyzed. The FFT-LSTM model [22], which replaces the time series data with the phase of the complex number using FFT, shows a slight improvement in accuracy; however, this improvement is minimal. The DeepAR model [44] and the Transformer model [36] yield somewhat better results. A significant reduction in error is observed with the FFT(1)-Transformer model. As in the case of [22], the approach of transitioning from the time domain to the frequency domain during time series forecasting has proven effective. Notably, the proposed approach achieved the best results, reducing the MASE error by 1.6%. This conclusion is supported by all other performance metrics used. All metrics for the first dataset are represented in Table 2.

**Figure 3:**
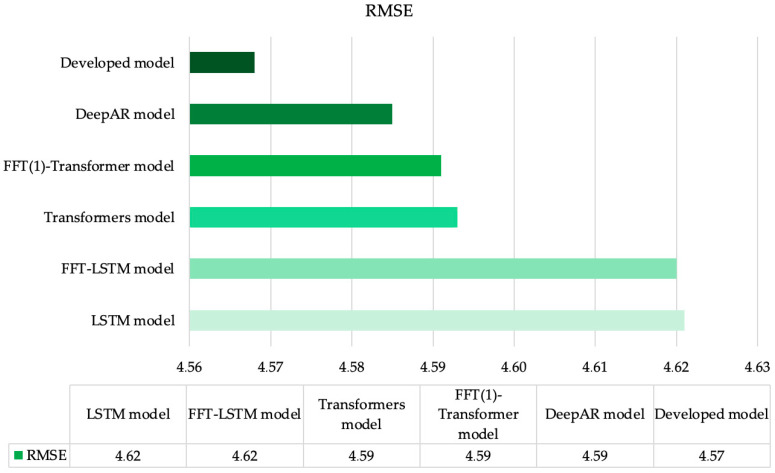
RMSE values for all investigated models using Dataset 2.

Figure 3 presents the accuracy comparison of all investigated models, illustrating RMSE errors for the second dataset. Once again, the LSTM model [43] demonstrates the lowest accuracy for this task. The FFT-LSTM model [22] slightly reduces forecasting errors, similar to the previous dataset. The Transformer model [23] and its enhancement, the FFT(1)-Transformer model [35], along with the DeepAR model [44], show a substantial decrease in error compared to earlier methods. The highest accuracy during the analysis of the second dataset is achieved using the method proposed in this paper, with the results corroborated by all other performance metrics utilized.

All metrics for the second dataset are represented in Table 3.

**Figure 4:**
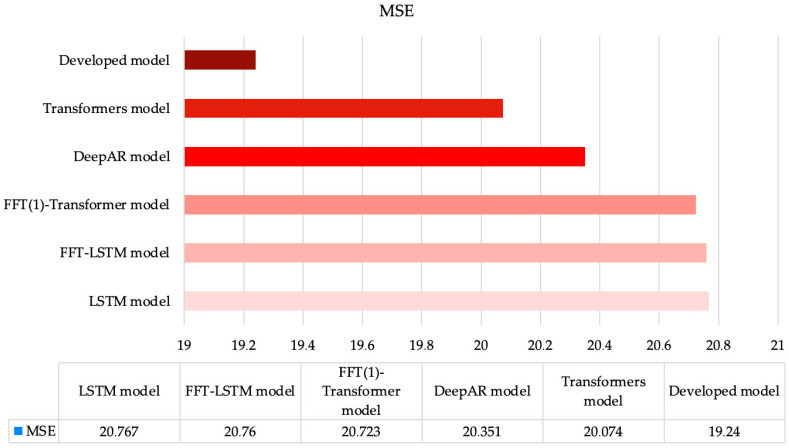
MSE values for all investigated models using Dataset 3.

Figure 4 presents the accuracy comparison of all investigated models, showcasing MSE errors for the third dataset. Here, the results are similar to the previous two cases, except for the models from [35,44]. Notably, the basic transformer architecture from [23] demonstrates better accuracy compared to its modified version using FFT-based preprocessing. This intriguing result may be attributed to the fact that transitioning from the time domain to the frequency domain using only the amplitude of the complex number may not provide sufficient information for this type of neural network architecture.

The proposed model in this paper, which achieves the highest forecasting accuracy among all methods analyzed for the third dataset, incorporates both the amplitude and phase of the complex number. While this approach effectively doubles the dimensionality of the input dataset, it also provides additional information to the neural network, resulting in superior performance. Specifically, the use of the proposed model reduces the MSE error of the closest existing method (the DeepAR model [44]) by over 4%. This conclusion is further supported by other performance metrics utilized in the analysis.

All metrics for third dataset represented in Table 4.

We also examined the computational characteristics of all the models under investigation. Specifically, we assessed factors such as the number of training parameters and the average training time across different datasets. The results, summarized in Table 4, provide an overview of the computational efficiency of each model. The average training time is reported in minutes, with all experiments conducted on an Nvidia T4 GPU to ensure consistency in performance evaluation. This analysis helps to highlight the trade-offs between model complexity and computational demands, offering insights into the practicality of deploying these models in real-world scenarios.

From the results presented in Table 5, it is evident that the methods studied differ in the number of training parameters. The FFT(1)-Transformer model has a considerable number of parameters (2,744,347), while both the Transformer model and DeepAR model have 2,556,847 parameters each. The LSTM and FFT-LSTM models each have 3,215,360 parameters, indicating greater computational complexity compared to the other models under investigation. Despite a significant expansion of the input data space, the proposed model only slightly increased the number of training parameters.

In addition, the training time for all the models studied varies significantly. DeepAR demonstrates the shortest average training time (140 min), making it the most computationally efficient model. The LSTM and FFT-LSTM models require the longest training time, 190 and 195 min, respectively. This indicates that these models are the most computationally expensive and require more resources and time to achieve comparable results. The training time for the proposed model, despite having somewhat more parameters compared to [35], is not the longest (180 min), which may suggest that its architecture is optimized for more efficient processing. Models with a larger number of parameters, such as LSTM and FFT-LSTM, require more training time, illustrating the need to balance model complexity against computational costs and performance accuracy.

In summary, the results of the modeling conducted on three datasets lead to the following conclusions:

- The employed deep learning neural network architectures provide good accuracy in solving time series forecasting tasks based on the analysis of large datasets;
- Transitioning from the time to the frequency domain through preprocessing methods, particularly using FFT, by replacing time series intervals with the phase of complex numbers, followed by the application of forecasting methods on such data, enhances forecasting accuracy using artificial intelligence. This is evidenced by both the Transformer model and the LSTM model across all three datasets. However, as shown by the results of experimental modeling on the third dataset, relying solely on the real part of the complex number may not provide enough information for the transformer architecture;
- The proposed preprocessing method utilizing FFT, which substitutes time series intervals with the phase and amplitude of complex numbers, achieves the highest forecasting accuracy compared to all models investigated in this study. This is attributed to the additional informative value of the new data fed into the neural network, considering not only the phase but also the amplitude of the complex numbers obtained from FFT;
- The developed model does not require the largest number of parameter adjustments; nevertheless, its training time is not the longest among the models considered. As a result, it achieves an optimal balance between training efficiency and accuracy, making it the ideal choice for applications that demand both high accuracy and satisfactory data processing speed;
- According to the results of the Diebold–Mariano test, the developed model exhibits a statistically significant improvement in predictive accuracy compared to the baseline model across all datasets.

Among the drawbacks of the developed model is the duplication of input data fed into the neural network due to the use of both phase and amplitude of complex numbers instead of the original time intervals. The perspectives for future research should include the following directions:

- Create and investigate the effectiveness of a preprocessing scheme based on feature extraction that incorporates the proposed approach and time series extension by adding the phase and amplitude of complex numbers to time intervals. This method can enhance the informational value of the neural network by combining time series data with their frequency characteristics, potentially increasing the forecasting accuracy of similar methods;
- Examine the effectiveness of preprocessing methods for time series that transition from the time domain to the frequency domain using wavelets, to achieve improved forecasting accuracy;
- Design an ensemble model for time series analysis through the combined use of FFT and wavelets for preprocessing, followed by the parallel processing of two or more new datasets using different or identical deep learning neural network architectures. The result aggregation mechanism could range from simple averaging to employing a meta-model to process the output signals from two or more first-level neural networks. While this approach may require significant computational resources, it has the potential to substantially improve forecasting accuracy, which may be critical for specific application tasks.

## 6. Conclusions

The amount and diversity of data collected by modern sensor networks and IoT systems are expanding rapidly. The effectiveness of tasks like time series forecasting for data collected by various sensors directly impacts decision making across a wide range of industries. While modern neural network approaches such as LSTM and transformers have demonstrated promising results, their performance is often hindered by challenges such as large data volumes, difficulties in capturing long-term dependencies, and the presence of noise and anomalies in the data. These challenges highlight the need for new solutions to enhance forecasting accuracy.

This article presents a novel approach to improve time series forecasting accuracy through the adaptation of transformer architecture and the introduction of a new data preprocessing method based on the fast Fourier transform (FFT). This approach transitions from the time domain to the frequency domain by replacing time intervals with the amplitude and phase of complex numbers, thereby increasing the informational value of the data.

Modeling on three different datasets confirmed that using FFT for data preprocessing yields higher forecasting accuracy compared to traditional methods. The study demonstrated that integrating phase and amplitude information significantly enhances results, particularly when employing transformers.

Among the drawbacks of the developed model is the redundancy of input data due to the use of both phase and amplitude of complex numbers, which may be acceptable when high forecasting accuracy is prioritized. Future research prospects include developing preprocessing schemes based on feature extraction techniques, particularly those utilizing wavelets, as well as creating ensemble models that could substantially improve forecasting accuracy.

## Figures and Tables

**Table 1:** Performance results of the proposed model utilizing three distinct datasets.

Performance Indicator/Dataset Number	Dataset 1	Dataset 2	Dataset 3
MASE	0.916	1.507	1.004
SMAPE	0.675	0.655	0.647
MAE	3.014	4.768	4.335
MSE	12.767	22.783	19.24
RMSE	3.563	4.568	4.98
Context size	60	24	60

**Table 2:** Performance results of the models on Dataset 1.

Performance Indicator/Dataset Number	Developed Model	FFT(1)-Transformer	Transformer	DeepAR	FFT-LSTM	LSTM	Naive
MASE	0.916	0.931	0.954	0.964	0.971	0.976	1
SMAPE	0.675	0.686	0.705	0.752	0.76	0.786	0.934
MAE	3.014	3.046	3.071	3.203	3.346	3.371	3.712
MSE	12.767	12.83	12.955	13.83	14.178	14.354	14.945
RMSE	3.563	3.581	3.599	3.718	3.765	3.788	3.973
Diebold–Mariano test	base	significant	significant	significant	significant	significant	significant

**Table 3:** Performance results of the models on Dataset 2.

Performance Indicator/Dataset Number	Developed Model	FFT(1)-Transformer	Transformer	DeepAR	FFT-LSTM	LSTM	Naive
MASE	1.507	1.624	1.573	1.617	1.725	1.715	1
SMAPE	0.655	0.754	0.703	0.73	0.79	0.784	0.792
MAE	4.768	4.806	4.792	4.782	4.82	4.812	4.856
MSE	22.783	23.293	23.33	23.286	23.351	23.348	23.847
RMSE	4.568	4.591	4.593	4.585	4.623	4.621	4.882
Diebold–Mariano test	base	significant	significant	significant	significant	siginificant	significant

**Table 4:** Performance results of the models on Dataset 3.

Performance Indicator/Dataset Number	Developed Model	FFT(1)-Transformer	Transformer	DeepAR	FFT-LSTM	LSTM	Naive
MASE	1.004	1.087	1.063	1.084	1.181	1.186	1
SMAPE	0.647	0.723	0.7	0.718	0.747	0.749	0.743
MAE	4.335	4.389	4.307	4.374	4.407	4.413	4.489
MSE	19.24	20.723	20.074	20.351	20.758	20.767	20.984
RMSE	4.98	5.576	5.148	5.543	5.599	5.604	5.631
Diebold–Mariano test	base	significant	significant	significant	significant	significant	significant

**Table 5:** Computational characteristics of all investigated models.

Computational Characteristic	Developed Model	FFT(1)-Transformer	Transformer	DeepAR	FFT-LSTM	LSTM
Number of trainable parameters	2,744,347	2,556,847	2,556,847	2,046,800	3,215,360	3,215,360
Average training time across datasets in minutes on Nvidia T4 GPU	180	165	165	140	190	195

## Data Availability

Publicly available datasets were used in this study. These data can be found here: [39,40,41].
